# Biological Roles of Lipocalins in Chemical Communication, Reproduction, and Regulation of Microbiota

**DOI:** 10.3389/fphys.2021.740006

**Published:** 2021-09-14

**Authors:** Romana Stopková, Tereza Otčenášková, Tereza Matějková, Barbora Kuntová, Pavel Stopka

**Affiliations:** Department of Zoology, Faculty of Science, Charles University, BIOCEV, Prague, Czechia

**Keywords:** lipocalins, odorant, mouse, major urinary protein, odorant-binding protein, retinol-binding protein, LCN, microbiota

## Abstract

Major evolutionary transitions were always accompanied by genetic remodelling of phenotypic traits. For example, the vertebrate transition from water to land was accompanied by rapid evolution of olfactory receptors and by the expansion of genes encoding lipocalins, which – due to their transporting functions – represent an important interface between the external and internal organic world of an individual and also within an individual. Similarly, some lipocalin genes were lost along other genes when this transition went in the opposite direction leading, for example, to cetaceans. In terrestrial vertebrates, lipocalins are involved in the transport of lipophilic substances, chemical signalling, odour reception, antimicrobial defence and background odour clearance during ventilation. Many ancestral lipocalins have clear physiological functions across the vertebrate taxa while many other have – due to pleiotropic effects of their genes – multiple or complementary functions within the body homeostasis and development. The aim of this review is to deconstruct the physiological functions of lipocalins in light of current OMICs techniques. We concentrated on major findings in the house mouse in comparison to other model taxa (e.g., voles, humans, and birds) in which all or most coding genes within their genomes were repeatedly sequenced and their annotations are sufficiently informative.

## Introduction

**Figure 1:**
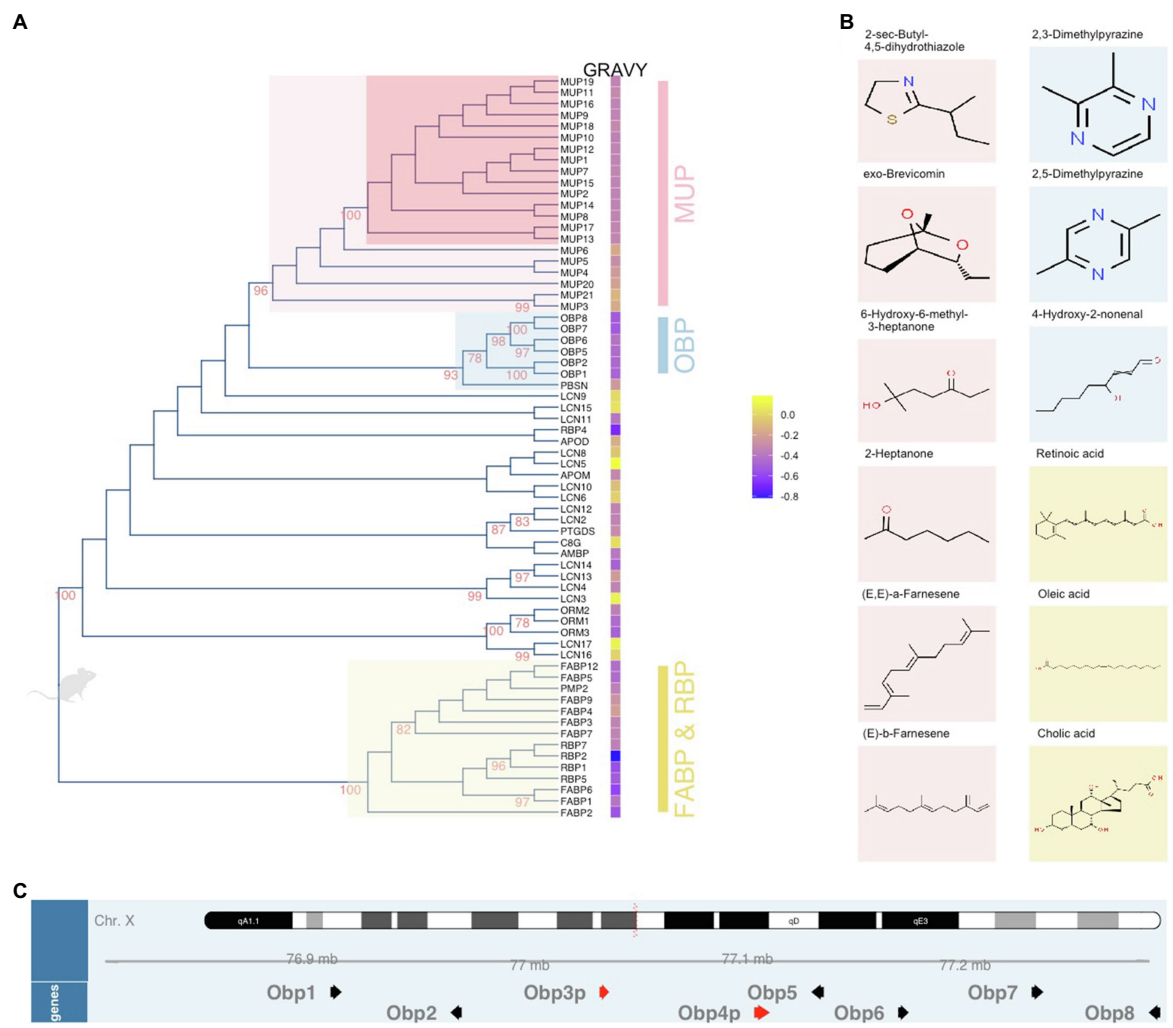
Unrooted dendrogram of mouse lipocalins within the Calycin superfamily. The evolutionary history was inferred by using the Maximum Likelihood method based on the JTT matrix-based model. The tree **(A)** with the highest log likelihood (−17,639.2427) is shown. The percentage of trees in which the associated proteins clustered together is shown next to the branches. Initial tree(s) for the heuristic search were obtained by applying the Neighbor-Joining method to a matrix of pairwise distances estimated using a JTT model. The tree is drawn to scale, with branch lengths measured in the number of substitutions per site. The analysis involved 66 amino acid sequences. There were a total of 406 positions in the final dataset. Evolutionary analyses were conducted in MEGA6 while the final figure was plotted with *ggtree*. Next to the tree, Gravy index is scaled from blue (hydrophilic) to yellow (hydrophobic). The colour code of highlighted subtrees is also used **(B)** to highlight the most common ligands with high affinities to particular protein groups. The position of Obp genes (black arrows) and pseudogenes (red arrows) on the X chromosome is visualized in **(C)** using Gviz Bioconductor package.

Initial mouse genome sequencing (Abril et al., 2002), re-sequencing of wild derived mice and other laboratory strains (Chang et al., 2017; Lilue et al., 2018) and now widely used OMICs techniques helped to link genotypes and phenotypes and provided new pieces of evidence that lipocalins are essential for life for their capacity to bind and transport biologically active as well as the toxic organic compounds in many fitness-related contexts. The lipocalins are small soluble proteins (typically 20kDa) with an eight-stranded antiparallel β-barrel often with two α-helices (N-terminal, C-terminal) on both ends of the protein, reviewed in (Stopková et al., 2009, 2014; Phelan et al., 2014b). The structure of the lipocalin β-barrel is open at one end allowing for the binding of various hydrophobic substances. There is yet another group of highly similar proteins that have β-barrels, though 10-stranded, and they include cellular retinol-binding proteins (CRBPs, depicted in Figure 1A as RBP1,2,5,7) and fatty acid binding proteins. The exception is extracellular RBP4 which, similarly as other lipocalins, have eight-stranded β-barrel. All together these proteins and lipocalins form the calycin superfamily (Sanchez et al., 2003; Álvarez et al., 2005). The lipocalin family is diverse accounting for at least 55 genes in the house mouse (Stopková et al., 2014) with little DNA sequence homology but with conserved tertiary structure thus forming β-barrel in all the mouse lipocalins but also in all metazoans over the evolutionary history (Ganfornina et al., 2000, 2006; Sanchez et al., 2003, 2006). Another interesting feature of lipocalins is their ability to bind and transport a wide spectrum of compounds including potentially toxic metabolic end-products such as ROS (reactive oxygen species) and also xenobiotics in many bacterial, plant, insect, avian, and mammalian species (Charron and Sarhan, 2006; Kwak et al., 2011, 2016; Bhatia et al., 2012).

From the evolutionary point of view, ancestral lipocalins can be found in many animal taxa including bacteria. The closest homologues of bacterial lipocalins are lazarillo in insects or vertebrate apolipoproteinD – APOD (Ganfornina et al., 1995, 2008; Bishop et al., 2006). Concerning the vertebrate calycins (incl. lipocalins), some members were found through all the taxa (fish, amphibians, birds, and mammals), for example, APOD, FABPs, C8G, AMBP, LCNs (Bilkova et al., 2018), while others are highly specific for mammalian species – major urinary protein (MUP), odorant-binding protein (OBP). Although many OBPs were detected in insect species, where they are involved in chemical communication, being highly abundant in antennae and having specific affinities to insect pheromones, there is no true homology between the vertebrate and insect OBPs (Pelosi et al., 2006; Vieira and Rozas, 2011). This supports the view that the radiation of OBPs happened independently during the evolutionary history.

The aim of this review is to further explore what features make the lipocalins essential for life from the mouse perspective but also from other vertebrates which makes the view on lipocalin functions more robust. Thus, in this review, we aim to extract biologically relevant features typical for different lipocalin proteins in the mouse based on amino-acid sequence similarities using a dendrogram (Figure 1A) that was generated from public data^1^ and using other characteristics and expression sites of particular lipocalins. In addition, we also re-opened and explored proteomic datasets from our previous experiments that are also publicly available to discuss them in light of other studies published to date. We discuss the importance of lipocalins in three major biological areas in which lipocalins play crucial roles, namely in chemical communication, reproduction and development, and in the regulation of pathogens and natural microbiota.

## Chemical Communication

Chemical communication is facilitated by behaviour-guiding olfactory signals, which in mice are abundant in body secretions such as tears, saliva, vaginal secretions, and urine (Thoß et al., 2015, 2016; Stopka et al., 2016; Cerna et al., 2017; Stopkova et al., 2017). These signals are often complex and provide information about multiple states of an individual including (sub-)species, kin, sex, health, and food sources (Hurst et al., 1997, 1998; Zala et al., 2004, 2015; Cheetham et al., 2007; Stopková et al., 2007; Bímová et al., 2009). Different types of molecules manifest the complexity of such signals which are detected *via* chemosensory G-protein coupled receptors of the main olfactory epithelia (MOE) and of the vomeronasal organ (VNO; Moss et al., 1997; Leinders-Zufall et al., 2000; Spehr et al., 2006; Wynn et al., 2012; Ibarra-Soria et al., 2014a; Nagel et al., 2018; Santoro and Jakob, 2018; Van Der Linden et al., 2018; Miller et al., 2020; Bansal et al., 2021). These receptors are tuned to particular signalling molecules including the volatile organic compounds – VOCs (Berghard and Buck, 1996; Malnic et al., 1999; Kwak et al., 2012, 2013), short peptides (Leinders-Zufall et al., 2004; Sturm et al., 2013) and non-volatile lipocalins, in mice, dominated by the male-biased MUPs (Chamero et al., 2007; Roberts et al., 2012). MUP like signals even have interspecific effects *via* the parallel mechanisms for kairomone detection. For example, lipocalins released from predators (cats, rats) are detected by mice and this induces aversive responses (Papes et al., 2010; Carvalho et al., 2015; Pérez-Gómez et al., 2015). Together, these secretory signals form signature mixtures, which generate stable representations in the accessory olfactory bulb (AOB) and, in other parts of the brain, these representations are linked to relevant behaviours (Bansal et al., 2021).

Major urinary proteins are essential for proper delivery of various volatile signals out of the body and for protecting and slowly releasing them from the urine marks (Hurst et al., 1998; Drickamer, 2001; Sharrow et al., 2002, 2003). Many of the ligands released from MUPs or just from the drying urine are directly detected by VNO neurons (Leinders-Zufall et al., 2000). MUPs were also demonstrated as signals themselves that are detected by basal, V2R-expressing sensory neurons in the VNO (Chamero et al., 2007) which due to small but detectable differences between different Mup genes and MUP protein structures (Phelan et al., 2014a,b) provide the basis for combinatorial coding of MUP signalling (Chamero et al., 2007; Kaur et al., 2014) which are then responsible for the display of specific mouse behaviours (Roberts et al., 2010, 2012; Dey et al., 2015; Demir et al., 2020). Importantly, these neurons are different from those that detect small hydrophobic compounds, which are apical V1R-expressing VNO neurons (Leinders-Zufall et al., 2000). In Figure 1A, MUPs are depicted in dendrogram as monophyletic group in which the associated proteins highly cluster together (bootstrap, BT=96). This group can be further separated onto sub-recently duplicated and highly similar “central” MUPs (BT=100) and the ancestral “outlier” MUPs 3, 4, 5, 6, 20, and 21 (Logan et al., 2008; Stopková et al., 2009, 2014; Phelan et al., 2014b; Sheehan et al., 2019). In Figure 1A, the Gravy index^2^ is provided in heatmap next to the protein names indicating that both groups of MUPs have different numbers of hydrophobic residua and thus the affinity to bind different VOCs; this difference is significant while their isoelectric points are similarly acidic pI=~4.9 (Stopková et al., 2016). This evolutionary differentiation of mouse MUPs has widened the spectrum of hydrophobic compounds that they protect, transport and release. This step was presumably crucial for the evolution of the house mouse as it enabled to “utilize” a wider and more informative odour space in individual recognition. Examples of typical MUP ligands (VOCs) previously studied in mice (Cavaggioni et al., 1987; Novotny et al., 1999; Zidek et al., 1999; Sharrow et al., 2003; Bingham et al., 2004) are presented in Figure 1B. For their complexity (volatiles, non-volatile proteins, and peptides), body secretions are now seen as signature mixtures rather than pheromones (Brennan and Kendrick, 2006; Nagel et al., 2018; Roberts et al., 2018; Bansal et al., 2021).

The system of signal detection has also diversified within the vertebrates thus evolving clear morphological structures (VNO, MOE) in tetrapods (except birds) while genetic components (V1R, V2R vomeronasal receptors) have already been found in teleost fish and sharks (Grus and Zhang, 2008). The transition from water to land was accompanied by a change in the ratio of V1R and V2R receptors such that the terrestrial vertebrates have more V1R receptors detecting smaller molecules including volatiles, whilst aquatic vertebrates have more V2R receptors for soluble and larger molecules including peptides and proteins. For example, amphibians (xenopus) have 21 V1Rs and 330 V2Rs, reptiles (python) have 4 V1Rs and 216 V2Rs, while the mouse system of olfaction is highly specialized for terrestrial life with 239 V1Rs (Tirindelli, 2021) with distinct evolutionary trajectories across mouse species (Miller et al., 2020), and 122 V2Rs (Tirindelli, 2021). Humans have lost functional VNO and the genes with remaining five intact V1Rs (Shi and Zhang, 2007). Profound differentiation of VNO and MOE in mice resulted in a highly specialized system being able to detect a wide spectrum of compounds including soluble (V2R) and volatile (V1R) compounds with VNO and general odorants with MOE. The signals detected by VNO are then processed in the AOB which maintain stereotypic sensory representations for broad types of stimuli, providing a substrate for relevant behaviours (Bansal et al., 2021). The responses of VNO and MOE are fast because odours are transported in turbulent plumes from the sites of origin and for example mice are able to detect these dynamically changing odours with a frequency of up to 40Hz (Ackels et al., 2021). The main message from this study is that volatiles, released from MUPs in the urine marks, must directly interact with chemosensory receptors and not *via* nasal volatile-binding proteins that would slow down this interaction. This is also suggested in a study on elephants, who produce the OBPs along the whole trunks making it unlikely to fast transport the volatile signals to the vicinity of chemosensory receptors from distant trunk parts (Lazar et al., 2002).

The most interesting aspect of olfactory diversification is a co-evolutionary process during which the receptor diversification was accompanied by the duplication and neo-functionalization of lipocalins. Particularly, there is a group of highly similar lipocalins (LCN3, LCN4, LCN13, and LCN14) which cluster together (BT=99, Figure 1A) and which are predominantly expressed in the mouse VNO thus forming of up to 36% of all transcripts in males and almost 29% in females with *Lcn14* being the most abundant transcript in VNO (Kuntova et al., 2018). Transcriptomic analysis of MOE provided similar results as what we have detected with proteomics of the nostrils with prevailing OBPs (*Obp2*, *1*, *5*, *8*, and *Mup4* transcripts). Interestingly, a total of 17 lipocalins were detected on the proteomic level in the mouse saliva including LCNs that were detected mainly in VNO (Stopka et al., 2016). Many of these proteins are presumably transported to the oral cavity by a system of tiny tunnels called naso-palatinal ducts which are required for proper pheromone signalling and functioning of VNO which is why they are concentrated near the VNO opening (Levy et al., 2020). It is likely that after the signals are detected in VNO, they are chelated by the VNO-specific LCNs with larger barrels and consequently pumped out and “released” to the oral cavity where they have been detected (Stopka et al., 2016) and where the digestion begins.

**Figure 2:**
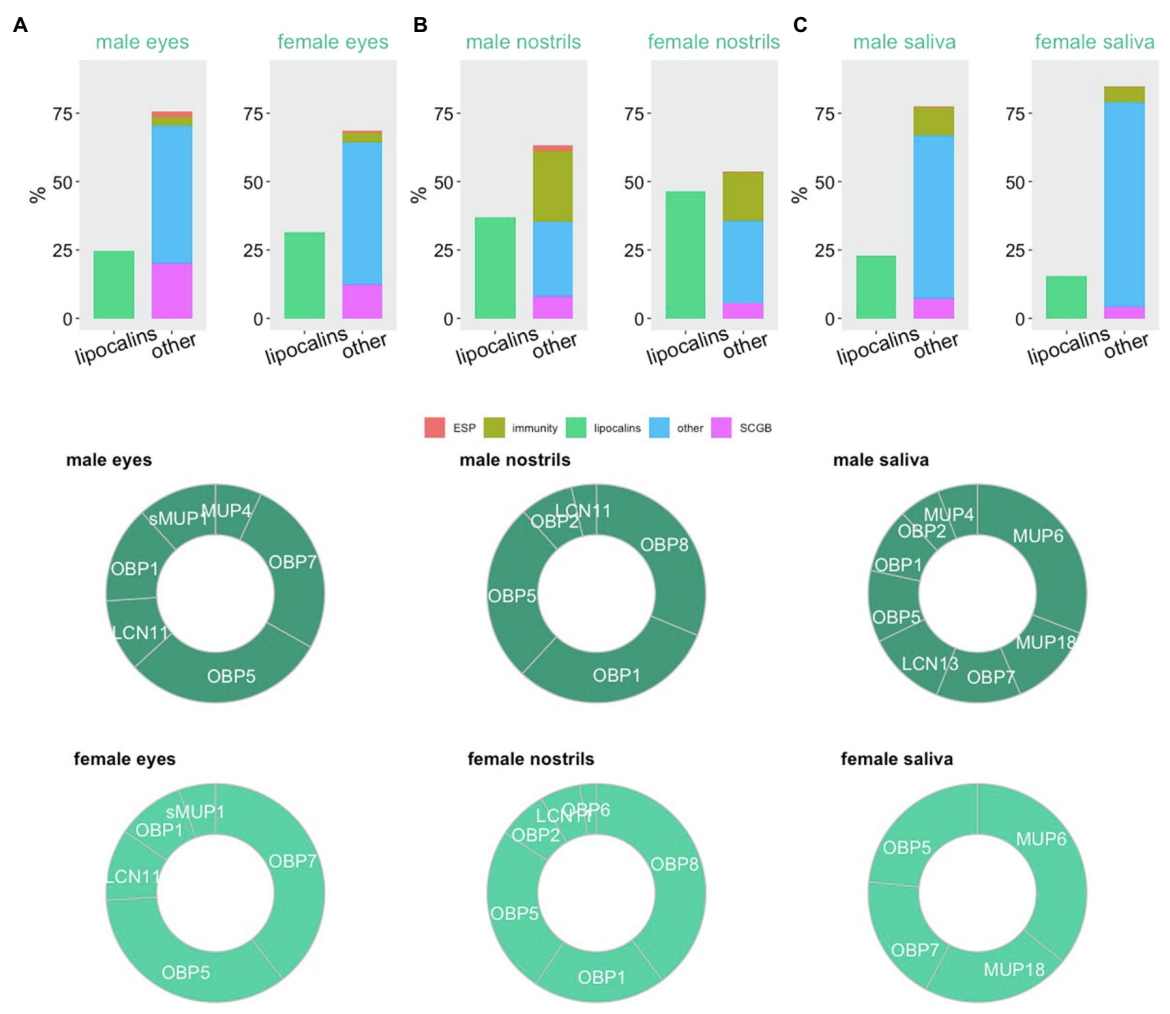
Proteomic signatures in major orofacial secretions of the house mouse. Estimation of protein abundances is represented as a fraction (%) of all proteins in male and female eyes **(A)**, nostrils **(B)**, and oral cavity **(C)**. We have extracted this data from our previously published studies with publically available data. Proxy for functions (exocrine-gland secreted peptides – ESP, immunity, lipocalins, secretoglobins – SCGB) is based on public gene ontology tool.

In mice, the neo-functionalization of lipocalins can be demonstrated by specific proteomic signatures, which in our case are presented as particular differences in the relative contribution to total protein content across different secretions. In Figures 2A–C,^3^ we present the data from our previous experiments where we generated the whole proteomes from lavages of eyes (Stopkova et al., 2017), nostrils (Kuntova et al., 2018), and oral cavity (Stopka et al., 2016) using label-free proteomics. We used just a few proxies to see relevant signatures, namely lipocalins, proteins involved in immunity and antimicrobial defence, secretoglobins [SCGB or ABP – androgen-binding proteins, formerly suggested as putative semiochemicals (Bimova et al., 2011)], exocrine-gland secreted peptides (ESP, putative pheromones; Haga et al., 2010; Abe and Touhara, 2014), and many other proteins with structural, homeostatic or cellular functions depicted as ‘other’. The most interesting result of this simple comparison is that the nostrils contain excessive amounts of lipocalins. In comparison with the urine which contains as much as 85% of lipocalins, mainly MUPs (Enk et al., 2016), the nostrils contain mainly the odorant binding proteins OBP8, OBP1, OBP5, OBP2 and a lipocalin named LCN11, Figure 2B.

The family of OBPs is coded by genes on the mouse chromosome X. To extend the knowledge on wild-living mice, we have sequenced all *Obp* transcripts and provided unique *Obp* sequences for feral *M. m. domesticus* (*Obp1* – KJ605385, *Obp2* – KJ605386, *Obp5* – KJ605387, *Obp6* – KJ605388, and *Obp7* – KJ605389), and *Mus musculus musculus* (*Obp1* – KJ605390, *Obp2* – KJ605391, *Obp5* – KJ605392, *Obp6* – KJ605393, and *Obp7* – KJ605394) now available in GenBank (Stopková et al., 2016). All the novel OBPs have a feature typical for the entire *Obp* cluster – a specific disulfide bond (Cys38–Cys42), which represents a strong OBP-diagnostic motif CXXXC (Cys-Xaa-Xaa-Xaa-Cys). OBPs are phylogenetically close to MUPs, Figure 1A. However, their biochemical properties are different from those of acidic MUPs in that their isoelectric points are closer to neutral and their hydropathy (Gravy) index is lower (Stopková et al., 2016). Thus, their affinity for less hydrophobic compounds, their prevalence in the proteomic profiles of eyes and nostrils and their close-to-neutral pI suggests that these proteins are important in removing the background organic molecules including odorants that are different from volatiles presented by MUPs in the urine. From the point of view of the “odour space” a fraction of odours and other compounds is being instantly removed from the nostrils and this presumably facilitates that the receptors preferentially bind relevant signals.

There is yet another perspective in the understanding of potential roles of OBPs in the mouse nostrils. They are sexually dimorphic similarly as the semiochemical responsive olfactory neurons (Vihani et al., 2020). This means that for some reason, being most likely a sharper detection of the ligands produced by males, females produce more OBPs (almost 50% of all soluble proteins) in the nostrils, which is likely to remove those VOCs that do not have the chemical properties of MUP ligands that males use for signalling. Similar pattern was detected in the eyes with similar levels of sexual dimorphism, Figure 2A. Because, eyes, nostrils and oral cavity are the inter-connected structures, with eyes being connected with nostrils *via* naso-lacrimal ducts, nostrils (VNO) with the oral cavity by the naso-palatinal ducts (Levy et al., 2020) and through naso-pharyngeal upper airways, OBPs may help removing the given spectrum of selected compounds from the majority of oro-facial mucosa. The selective forces that shaped the evolution of OBPs probably included the ability to remove organic toxins from nasal epithelia and upper airways. This has been shown in porcine OBPs, which bind with high affinity HNE (4-hydroxy-2-nonenal) depicted in Figure 1B, a toxic compound derived from lipid peroxidation. OBPs therefore protect living cells from damage caused by oxidative stress (Grolli et al., 2006). In non-rodent taxa, such as Artiodactyla (Ruminantia), the olfactory secretome becomes more complex during the reproductive period. For example in goats and sheeps, more OBP variants are expressed and the ligand reception by OBPs is often facilitated by protein modifications including O-GlcNAcylation (Cann et al., 2019), which was previously described also in pigs (Nagnan-Le Meillour et al., 2014).

In many mammals, Obp genes were selected for diverse functions across various taxa. Arvicolids (Cricetidae) are a parallel lineage to Murid rodents (Muridae) within the superfamily Muroidea and they seem to lack the genes for MUPs. However, they produce excessive amounts of OBPs in their urine and several studies provided evidence that they are produced by the liver. For example, the bank voles (*Clethionomys glareolus*) produce at least three OBPs coded by Obp1, Obp2, and Obp3 genes encoding the predominant lipocalins present in their urine and saliva (Stopkova et al., 2010), see also (Loxley et al., 2017). Similarly, the European water voles (*Arvicola terrestris*) produce one sexually dimorphic OBP named arvicolin with transcripts being highly abundant in the liver and proteins present in the urine. The reception of pheromones by this particular OBP may be facilitated *via* protein modifications (e.g., phosphorylation and O-GlcNAcylation; Nagnan-Le Meillour et al., 2019) while the hamster OBP (aphrodisin) is *N*-glycosilated (Singer et al., 1986). All these arvicolid and also mouse OBPs are highly similar to hamster aphrodisin, which is vaginal OBP that was named after its effect upon male-mounting behavior *via* its natural OBP ligands (Singer et al., 1986; Briand et al., 2004) and is also expressed by other hamster species (Turton et al., 2010). When looking further from the muroid rodents (Myomorpha), OBPs have also been detected in the urine of highly social African mole rats (Hystricomorpha; Hagemeyer et al., 2011) thus providing evidence that the involvement of OBPs in chemical signaling was likely the ancestral mode of chemical communication in rodents. Taken together, the selective pressures that drove the evolution of chemical communication in rodents acted upon the function of a range of lipocalins and not on particular lipocalin gene. This is evidenced by the fact that those species that lack the genes for MUPs evolved other lipocalins for this function so the function of MUPs as carriers of chemical signals was supplanted by OBPs in many rodent taxa.

An interesting aspect of the systems of signalling and detection is that they are socially (environmentally) modulated. To our knowledge, we have been the first laboratory that provided evidence that the abundance of MUPs in the urine of female mice correlates with the estrous cycle, reaching the highest levels od MUP abundance in the urine (Stopka et al., 2007) and vaginal secretions (Cerna et al., 2017) near estrus. There is also a strong influence of social environment, whereby males of wild *M. m. musculus* increase their production of MUPs in the urine when presented with a female behind metal grid (Janotova and Stopka, 2011). Socially induced MUP variation has also been demonstrated in seminatural enclosures, where males doubled the excretion of MUPs after acquiring a territory and became socially dominant (Thoss et al., 2019). Higher concentrations of MUPs may then yield various behavioural responses in the receiver due to MUP detection by VNO neurons *via* progesterone signalling (Dey et al., 2015). Recent development of transcriptomic techniques helped to reveal that the fitness-related social dynamics of protein expression was also demonstrated in olfactory tissues. The expression of many receptor genes was altered when presented with different stimuli in several strains of the laboratory mice (Ibarra-Soria et al., 2017) and similarly when mice are separated, the isolation induces sex-specific differences in the olfactory sensory receptor repertoires (Santoro and Jakob, 2018; Van Der Linden et al., 2018). These social and reproductive effects are relevant examples of the expression dynamics that is regulated by benefits and costs of lipocalin and receptor production driven by social stimuli and the potential to mate. Until this point, we concentrated on lipocalins that function as an interface between external and internal worlds of an individual. However, many lipocalins have important functions within an individual homeostasis, reproduction and development.

## Roles of Lipocalins in Reproduction and Development

The production of fully mature and motile sperm cells is essential for successful reproduction. The first key event happens in the seminiferous tubules where, through a complex process of spermatogenesis, germ stem cells differentiate into highly polarized spermatozoa. Along testosterone, vitamin A in the form of retinoic acid (RA) is an important signalling molecule that initiates spermatogonial differentiation and meiotic entry (Zhou et al., 2008). After the conversion from retinol, which is synthetized in Sertoli cells, RA specifically binds to intracellular heterodimeric receptors RARs and RXRs and regulates gene expression, e.g., of Stra8 gene inducing the synthesis of downstream markers of meiosis (Livera et al., 2002; Zhou et al., 2008). Signalization mediated by RA and its receptors was shown to be important also for the normal function of steroidogenic Leydig cells producing testosterone (Jauregui et al., 2018). However, as retinoids are not soluble in an aqueous environment, they require both extracellular and intracellular binding proteins. Interestingly, lipocalin-type prostaglandin D_2_ synthase (L-PGDS or PTGDS) is expressed in many tissues, also including the mouse testis (Gerena et al., 2000). Since PTGDS has been reported to have a high affinity to RA and retinaldehyde, it was proposed that it might serve as a retinoid carrier supplying developing germ cells (Urade and Hayaishi, 2000; Rossitto et al., 2015). Moreover, Kido et al. (2005) showed the specific expression in transgenic mouse testis of fatty acid binding protein 9 (FABP9). Later study specified the FABP9 localization to advanced stages of spermiogenesis – from elongating spermatids on (Selvaraj et al., 2010). FABP9 shows high homology (58%) to FABP4, which binds long-chain fatty acids like stearate, palmitate, and oleate, but also RA, though with lower affinity (Oko and Morales, 1994; Richieri et al., 2000). Because spermatogenesis is a process demanding high levels of fatty acids (e.g., for membrane biogenesis), FABP9 likely contributes to providing them to the germ cells.

Fully developed testicular spermatozoa further undergo several maturation steps during their transit through epididymis, which is divided into three segments, each with unique luminal environment determined by proteins secreted from various epithelial cell types (Turner et al., 2003). The modification of sperm protein profile in region-specific manner is one of the proposed features of epididymal maturation. Support for this was provided by proteomic analysis of *M. musculus* whole sperm isolated from the caput, corpus and cauda regions (Skerget et al., 2015). The study showed that, among other proteins, lipocalins LCN2, LCN6, and LCN8 were downregulated in the caput sperm, whereas LCN12 was abundant in the corpus. On the contrary, LCN5 and FABP9 were common in sperm from all the segments. Moreover, Guyonnet et al. (2012) specified in CD 1 mice the FABP9, LCN5 and LCN12 localization to the matrix of sperm acrosome, while LCN2 and LCN6 signals were observed using fluorescent techniques to be associated with the mouse and human spermatozoa isolated from cauda epididymis (Chu et al., 2000; Hamil et al., 2003).

Despite the pieces of evidence, biological functions of many lipocalins in reproduction still remain unclear. LCN8 is hypothesized to bind and transfer retinoids (RA) similarly as LCN5 and thus might drive the development and maintenance of epididymal epithelium (Rankin et al., 1992; Costa et al., 1997; Lareyre et al., 2001). Based on an increased uptake of ferric ion by sperm mediated by LCN2, lipocalins are also suggested, by yet unknown mechanism, to undergo internalization into the sperm cell (Elangovan et al., 2004). In order to elucidate the role of lipocalins and cytosolic calycins in male reproduction, several knockout studies have been performed. Deletions of either of Lcn6, Lcn8, Lcn9, or Fabp9 did not cause abnormalities in the morphology of testis or epididymis nor in sperm production and fertility. However, the sperm from Lcn8^−/−^ and Fabp9^−/−^ double-knockout male mice showed abnormal head and tail morphology. An enhanced frequency of spontaneous acrosome reaction occurred in Lcn6 and Lcn8 deficient sperm (Selvaraj et al., 2010; Yin et al., 2018; Wen et al., 2021). All this evidence indicates that particular lipocalins are not essential for sperm development and its fertilizing ability because they can, to some extent, supplant each other. Very likely, it might be due to functional compensatory mechanism originating from their high structural and hence also binding homology. Therefore, double or triple knockout studies should be performed to undoubtedly elucidate this mechanism. The major significance of lipocalins in male reproduction thus remains in epididymal sperm maturation and events linked to capacitation and acrosome reaction occurring in the female reproductive tract.

**Figure 3:**
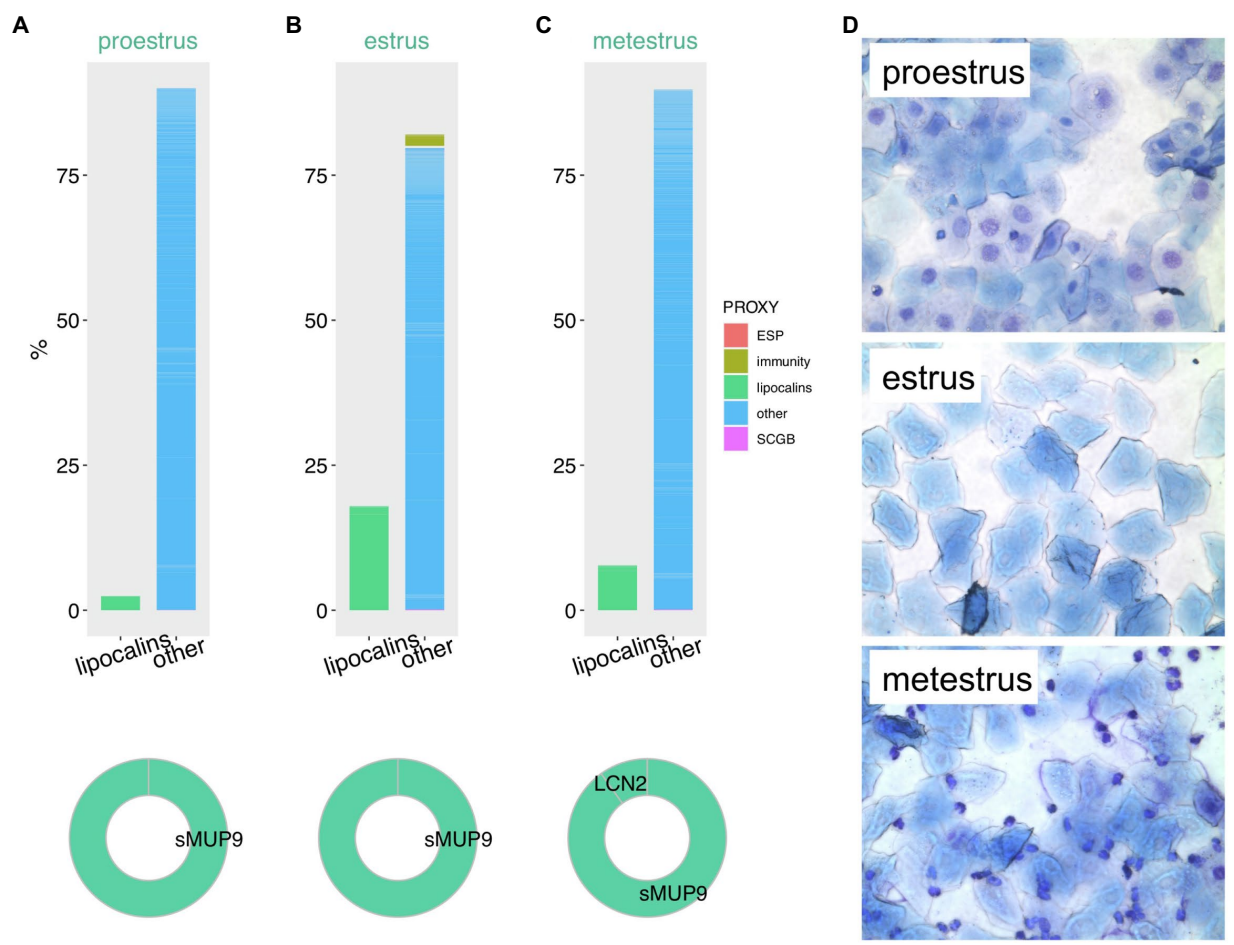
Proteomic variation in vaginal secretions throughout the mouse estrous cycle. Estimation of protein abundances is represented as a fraction (%) of all proteins in vaginal lavages during proestrus **(A)**, estrus **(B)**, and metestrus **(C)**. We have extracted this data from our previously published study with publically available data. Proxy for functions (ESP, immunity, lipocalins, secretoglobins – SCGB) is based on public gene ontology tool. Representative microphotographs of the vaginal cytology were taken at magnification 100× after May-Gruendewald and Giemsa staining **(D)**.

The estrous cycle along with the oogenesis, which is linked to folliculogenesis are the most important fitness-related processes in female reproduction. In mice and other rodents, the cycle is generally divided into the four phases known as proestrus, estrus, metestrus, and diestrus. Interestingly, in some rodents such as the wood mice (*Apodemus sylvaticus*) these phases dynamically change as a reaction to the presence or absence of male mating partners (Stopka and Macdonald, 1998). Particular phases are characterized by the differential gene expression and by varying cellular types of the utero-vaginal epithelia (Figure 3D) reflecting the maturation of ovarian follicles (Byers et al., 2012; Yip et al., 2013; Cora et al., 2015). In detail (see Figure 3D), the proestrus phase is typical with predominant nucleated epithelial cells, whereas estrus phase with keratinized epithelial cells on which bacteria feed. The metestrus is overpopulated with neutrophils. Hormonal imbalance or pathogen exposure may lead to disruption of cycling. As shown in Figures 3A–C using the vaginal fluid proteomic data from *M. m. musculus*, lipocalins are significantly up-regulated in estrus and metestrus compared to proestrus (Cerna et al., 2017). This stage-specific elevation was proportionally most obvious in sMUP9 (i.e., group of highly similar MUPs: MUP6, MUP9, MUP16, and MUP19) while LCN2 only mildly varied, Figure 3B, and was upregulated only in metestrus (Figure 3C). Except these, MUP20, sMUP17, LCN11, and OBP5 were significantly up-regulated in estrus but their proportion to total protein content was lower. On the contrary, retinol-binding protein 1 (RBP1) had the lowest abundances during estrus. Given their proposed ability to internalize and transport various ligands, highly abundant lipocalins in these stages possibly detoxify the mouse vaginal environment while some of these ligands might have become the signals by which males recognize female receptivity (Stopková et al., 2009).

High abundances of LCN2 limits the bacterial growth during estrus by iron-chelating bacterial siderophores (Flo et al., 2004). Similarly to male reproduction, retinoids are essential also for the female genital system. Vitamin A sufficiency is important for the differentiation of meiotic germ cells, embryonic implantation and normal embryonic development. The significance of the uterine expression of retinoid binding proteins and their transcripts, e.g., for RBP1, RBP4, CRABP2 thus supports this view (Clagett-Dame and Knutson, 2011; Yip et al., 2013). Additionally to the LCN2 protective roles for female genital-tract epithelia, this lipocalin also functions as a sperm capacitation-promoting factor *in vitro* (Watanabe et al., 2014). Isolated sperm from the oviduct of Lcn2^−/−^ knockout females showed a significant decrease in membrane lipid raft movement, which was retrieved after LCN2 addition. The association of LCN2 with sperm surface also enhances the progressive motility (Lee et al., 2003). The interaction between mammalian spermatozoa with seminal fluid, and female genital tract is known to be reciprocal. For instance, spermadhesins from ejaculate, apart from other functions, modulate the uterine immune responses. As lipocalins are contained in male genital fluid, they very likely co-participate on this direct interaction (Rodriguez-Martinez et al., 2010). To add, lipocalins are essential for various stages of reproduction both in males and females and similar lipocalins were detected also in avian ova (Bilkova et al., 2018), thus suggesting that the roles of lipocalins are conserved across distant vertebrate taxa.

## Regulation of Pathogens and Natural Microbiota

Lipocalins are expressed in all body parts with the complex microbial communities (Seo et al., 2006; Stopka et al., 2016; Cerna et al., 2017; Xiao et al., 2017) and their typical β-barrel structure with a central cavity for binding of various hydrophobic molecules (Flower, 1996) provides several strategies for constant sensing and controlling both pathogens and commensal bacteria. There is growing evidence that each microbiome-populated body site hosts unique bacterial ecosystems (Lee and Mazmanian, 2010; Matějková et al., 2020; Moudra et al., 2021) and that those tissues permanently face the challenge of discriminating between commensal and pathogenic bacteria. A rapid destruction of potential pathogens is especially important in the sites where pathogen burden could have detrimental effects upon the host. Such places include the oral cavity representing the main route to the digestive tract, urogenital openings, and eyes and nostrils as a gate to the brain *via* the optic and olfactory nerves (Stopková et al., 2014).

**Figure 4:**
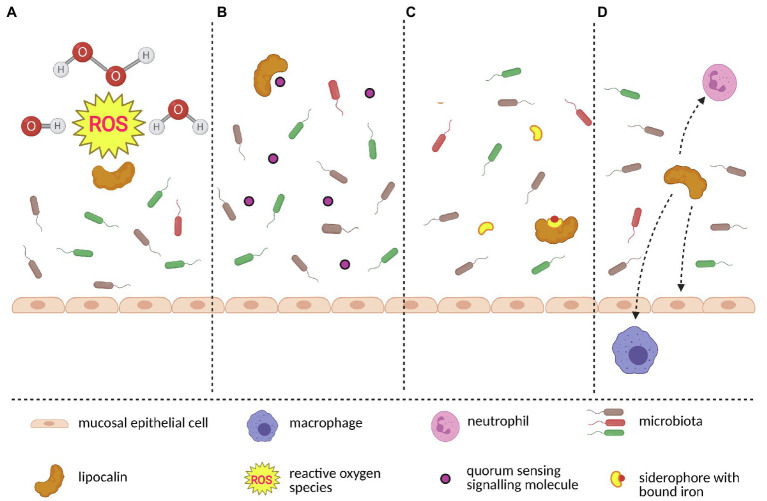
A model of interactions between lipocalins and microbiota. Lipocalins protect host’s epithelial cells and also commensal microbiota by scavenging toxic molecules such as reactive oxygen species which originate during oxidative stress **(A)**. Keeping microbiome homeostasis requires blocking the origin of bacterial monoculture. Lipocalins contribute to diversified microbiome by scavenging bacteria-produced quorum sensing signalling molecules **(B)**. Similarly, certain bacterial species use siderophores to gain iron and subseuently outcompete siderophore-lacking bacteria. By binding to siderophore-iron complex, lipocalins further contribute to stable microbiome **(C)**. Finally, lipocalins closely interact with other members of the immune system network (i.e., neutrophils, macrophages and epithelial cells) and together create a complex microbiota-surveillace system **(D)**. The figure was created in Biorender.

Communication between the host and its microbiome is facilitated by molecules secreted by bacteria. Microorganisms (and especially bacteria) belonging to the same species are able to synchronize their activities *via* so-called quorum sensing, Figure 4.^4^ Each quorum sensing is characterized by a signalling compound, usually a small molecule (QSM – quorum sensing molecule) which is released into the environment and recognized by other co-specific microorganisms (Rutherford and Bassler, 2012). When the concentration of QSM reaches a certain threshold, the whole population of microorganisms synchronizes its biosynthetic activity. QSMs can be inactivated by quorum quenching mechanism (QQ), which is usually mediated by specific enzymes (Uroz et al., 2009). However, the alternative strategy for QQ is the scavenging and removal of QSMs provided by the proteins with a QSM binding capacity. Ligands associated with bacterial infections and those from defeated bacteria during regulation of microbiota are also detected with MOE and VNO *via* the microorganism-associated molecular patterns (MAMPs), and they are sensed in many places in the body including specific chemosensory neurons in the mammalian nose (Bufe and Zufall, 2016). They also include the formyl peptide receptor-like proteins in VNO, which provide sensitivity to disease/inflammation-related ligands (Riviere et al., 2009) and presumably they are responsible for the activation of bactericidal proteins. Bactericidal proteins (i.e., such as BPI proteins) were previously detected in the olfactory transcriptomes of the mouse (Ibarra-Soria et al., 2014b) in tears (Stopkova et al., 2017) and saliva (Stopka et al., 2016).

Lipocalins have the ability to bind a broad spectrum of ligands, therefore it is likely that they are involved in the regulation of microbiota and of the function of olfactory neurons (Bryche et al., 2021). For example, a recent study provided the evidence that bovine (bOBP) and porcine (pOBP) odorant binding proteins can effectively bind farnesol, which is a terpenoid produced by *Candida albicans* as QSM and which is able to affect transformation from the mycelial to the yeast state (Bianchi et al., 2019). Moreover, both OBPs have the capability to almost completely remove bacterial toxin pyocyanin produced by *Pseudomonas aeruginosa*. To some extent, both OBPs have also the affinity to various types of *N*-acyl-homoserine lactones produced as QSMs by *P. aeruginosa* and some other microbial organisms. Despite the lower affinity of OBPs to *Pseudomonas* signalling molecules, OBPs might scavenge QSMs when they reach potentially dangerous concentrations that activate quorum sensing. Interestingly, assays testing the direct antimicrobial or antifungal activity revealed different efficiencies of the two OBPs. While pOBP has higher inhibitory activity on fungi, bOBP can better reduce the growth of various bacterial species (Bianchi et al., 2019). It is the first evidence that vertebrate odorant binding proteins exert antimicrobial activity through their scavenging capacity. Furthermore, OBPs also shape the microbiomes by scavenging toxic molecules (i.e., aldehyde 4-hydroxy-2-nonenal) that are produced during oxidative stress (Lacazette et al., 2000; Grolli et al., 2006) and whose higher concentrations cause damage not only to epithelial cells but also to commensal microbiota (Macedo-Marquez et al., 2014) which play a crucial role in keeping epithelial homeostasis (Koskinen et al., 2018).

Although the members of microbial communities are often described as commensals, the relationship between the host and a particular bacterial species can simply change to other forms of relationship as mutualistic or parasitic depending on the host’s genetic background, nutritional status, or co-infection of the host (Belkaid and Harrison, 2017). One of the main aspects influencing the bacterial switch from commensal to pathogen is the availability of iron, which is strongly regulated by lipocalins (Lechner et al., 2001; Goetz et al., 2002; Fluckinger et al., 2004; Julien et al., 2019). Iron is one of the essential nutrients for almost all aerobic organisms. In mammals, the majority of iron is bound either in hemoglobin or in ferritin and transferrin. Therefore, the concentration of extracellular free iron is maintained below 10^−24^M (Correnti and Strong, 2012). This low concentration of available iron serves as a protection of the host against the reactivity of free iron and also against the potentially pathogenic bacteria that would thrive in an iron-rich environment. To “steal” iron from host proteins (i.e., ferritin, transferrin, and lactoferrin), bacteria synthesize and secrete low-molecular-weight (<1.0kDa) iron chelators called siderophores (Nairz et al., 2010). One of the most studied siderophores is enterochelin (Ent) whose affinity for iron allows it to effectively outcompete the majority of the host’s iron-binding proteins (Fischbach et al., 2006). Typical lipocalin involved in iron homeostasis is LCN2 (alias 24p3, SIP24, siderocalin, NGAL), which is constantly present in low amounts in mucosa where it chelates Ent in order to inhibit overgrowing of Ent-producing bacteria. To prevent Ent from delivering iron back to the potential pathogens (e.g., to *Escherichia coli*, one of the major pathogens of gut-origin sepsis), LCN2 chelates the Ent-iron complex and forms a structure which is not capable of transferring iron back to bacteria (Goetz et al., 2002; Johnson et al., 2010; Wu et al., 2010; Mori et al., 2016). While LCN2-siderophore binding capacity is limited towards one group of siderophores – catecholate-type (Xiao et al., 2017), human tear lipocalin (LCN1) is capable of binding a broader array of siderophores, including the bacterial catecholate and hydroxamate-type, and all major classes of fungal siderophores (Fluckinger et al., 2004). This physiological function of LCN2 and human LCN1 is widespread in vertebrates and for example, in birds, four proteins highly homologous to LCN2 (CALβ, CALγ, Ggal-C8GC and Ex-FABP) exerted almost identical roles in the protection against *E. coli* (Garénaux et al., 2013).

Protective function of LCN2 is documented in experiments with Lcn2-deficient mice, which are highly prone to bacterial infection and sepsis. In detail, the lack of LCN2 leads to significant changes in gut microbiota composition followed by microbiota dysbiosis with a disproportionate growth of gram-negative bacteria (Moschen et al., 2016; Singh et al., 2016, 2020). Reducing effects on bacteria were observed in a study focused on *in vitro* growth of *Aeromonas hydrophila* and *E. coli* in the presence of fish LCN2 (ortholog – 3nLcn2; Zhou et al., 2020). Similarly to *in vitro* experiments, there were lower bacterial loads in tissues of 3nLcn2-administered fish infected by *A. hydrophila* (Zhou et al., 2020). Homeostatic relationships with microbiota can be kept only when the contact between bacteria and the host’s epithelial cells surface is minimized. This segregation of microbiomes from the host’s cells is accomplished by combined action of epithelial cells, mucus and the involvement of both innate and adaptive immune systems (Shi et al., 2017). The observation that Lcn2-deficient animals are also susceptible to the siderophore-independent pathogens suggests that LCN2 protects the microbiome-host barrier not only as a siderophore scavenger but also plays an important role in a broader immune system network.

## Conclusion

From the evolutionary point of view, lipocalins represent a fascinating protein family because, in vertebrates, many of their members underwent rapid evolution and neo-functionalization during or after the transition from water to land and this is well documented in rodents. At the same time, there is a group of lipocalins and other calycins that seem to be essential for life, because they are stereotypically expressed in almost all vertebrates and play important roles in spermatogenesis and embryonic development (FABP, RBP), regulation of oxidative stress (AMBP), and antimicrobial defence (e.g., LCN2). The transition from water to land was the major driving force that has driven the reorganization of sensory systems of detection (nose, eyes) of fitness-related cues such as food, predators and other individuals of the same species and particular sex. Mice are primarily dependent on olfactory cues. They use MUPs to protect and transport volatile pheromones that trigger many physiological, behavioural and reproductive responses *via* the vomeronasal and major olfactory systems. Various tissues in the oro-facial region, including lymphoid tissues, epithelia and lacrimal glands produce excessive amounts of OBPs which have different binding properties than MUPs (e.g., lower hydrophobicity) and thus they represent the major system of chelators of signals that are different from those presented by MUPs. In VNO these chelators include specific lipocalins LCN3, LCN4, LCN13, and LCN14, while MOE is overpopulated by OBPs. Interestingly, many lipocalins have similar barrels across the vertebrate taxa and thus they may have overlapping roles in tissue detoxification and in the regulation of bacterial growth. To conclude, for their capacity to bind various lipophilic and other organic molecules, lipocalins are essential for life, and this is documented by parallel evolution of their functions across animal taxa that inhabited the terrestrial ecosystems.

## Author Contributions

RS and PS wrote the first draft of the manuscript. TO prepared the involvement of lipocalins in reproduction while TM worked on the microbiota section. BK helped with discussions and analysis of literature searches. All authors contributed to the article and approved the submitted version.

## Funding

This research team was established as an output of MICOBION teaming project funded from EU H2020 (No. 810224). PS and TO were funded by the Czech Science Foundation (GAČR) project No: 19-22538S. TM was funded by the Grant Agency of the Charles University, Prague (GAUK; No. 1191419).

## Conflict of Interest

The authors declare that the research was conducted in the absence of any commercial or financial relationships that could be construed as a potential conflict of interest.

## Publisher’s Note

All claims expressed in this article are solely those of the authors and do not necessarily represent those of their affiliated organizations, or those of the publisher, the editors and the reviewers. Any product that may be evaluated in this article, or claim that may be made by its manufacturer, is not guaranteed or endorsed by the publisher.

## Footnotes

^1^ https://www.ensembl.org/

^2^ http://www.gravy-calculator.de/

^3^ http://string-db.org

^4^ https://app.biorender.com/
